# Sugar signals pedal the cell cycle!

**DOI:** 10.3389/fpls.2024.1354561

**Published:** 2024-03-18

**Authors:** Sanjay Singh Rawat, Ashverya Laxmi

**Affiliations:** National Institute of Plant Genome Research, Aruna Asaf Ali Marg, New Delhi, India

**Keywords:** target of rapamycin (TOR), SnRK1 kinase, CDK (cyclin-dependent kinase), sugar signalling, glucose

## Abstract

Cell cycle involves the sequential and reiterative progression of important events leading to cell division. Progression through a specific phase of the cell cycle is under the control of various factors. Since the cell cycle in multicellular eukaryotes responds to multiple extracellular mitogenic cues, its study in higher forms of life becomes all the more important. One such factor regulating cell cycle progression in plants is sugar signalling. Because the growth of organs depends on both cell growth and proliferation, sugars sensing and signalling are key control points linking sugar perception to regulation of downstream factors which facilitate these key developmental transitions. However, the basis of cell cycle control via sugars is intricate and demands exploration. This review deals with the information on sugar and TOR-SnRK1 signalling and how they manoeuvre various events of the cell cycle to ensure proper growth and development.

## Introduction

Energy derived from sugars propels a wide range of activities essential for an organism to function properly. This is orchestrated by cell signalling events that comprises the regulation of various proteins by upstream kinases and their downstream signalling effectors. Since cell division cycle in plants is responsive to energy availability, the role of sugars, such as glucose and sucrose, holds utmost importance in this regard, as they are the end products of photosynthesis. Sugars as signalling molecules are important re-modelers of an organism’s metabolism as well as physiology. The majority of the responses governed by sugars are mediated by the highly conserved serine/threonine kinase, TOR (TARGET OF RAPAMYCIN), the master regulator of key developmental processes in eukaryotic cells (Wullschleger et al., 2006; Dobrenel et al., 2013; Xiong and Sheen, 2015). On the contrary, their insufficiency induces an altogether different transcriptome through the AMPK (AMP-ACTIVATED PROTEIN KINASE)/SNF1 (SUCROSE NON-FERMENTING 1)/SnRK1 (SNF1-RELATED PROTEIN KINASE 1) signalling that favours stress induced responses over growth (Hedbacker and Carlson, 2008; Mihaylova and Shaw, 2011; Crozet et al., 2014; Margalha et al., 2019).

It was as early as 1966 when Van’t Hof demonstrated the provision of sucrose to excised pea root tips in promoting transition of cells from G_1_ to enter the active M phase (Hof, 1966). This was probably the first report on the crucial bearing of sugar signalling in the regulation of the cell cycle. Thereafter, several other studies have pointed towards the importance of sugars as crucial metabolites in the control of cell cycle. For instance, in the budding yeast *Saccharomyces cerevisiae*, fermentable sugars like glucose and sucrose activate protein kinase A (PKA) which is required for the expression of both growth and stress responsive genes, inactivation of which leads to arrest at the G_1_ phase of the cell cycle (Santangelo, 2006; Zaman et al., 2008). Similarly, in the fission yeast *Schizosaccharomyces pombe*, glucose depletion seems to reduce cell size and restrict the cells in the G_2_ phase (Masuda et al., 2016). Similar yet complex mechanisms exist in multicellular organisms in the regulation of cell cycle by various factors including nutrients, sugars, oxygen, amino acids etc (Elledge, 1996; Fingar and Blenis, 2004). Specifically, the TOR kinase integrates these signals to regulate cell growth and proliferation (Loewith and Hall, 2011). For instance, nutrients are particularly important TORC1 (TOR complex1) activator in unicellular organisms like yeast (Wang and Proud, 2009). Furthermore, more than one pathway is implicated in mTOR activation in mammals (Foster and Fingar, 2010). Additionally, the glucose-induction of mTOR (mechanistic/mammalian TOR) activity occurs in an amino acid (AA) dependent pathway, suggesting AA supremacy over other factors on mTOR activation (Avruch et al., 2009; Segev and Hay, 2012; Jewell et al., 2013). Interestingly, in plants, sugar-activation of TOR is a prerequisite to induce cell proliferation at the shoot and root meristems and lies upstream of its induction by other factors (Xiong et al., 2013; Pfeiffer et al., 2016; Li et al., 2017). Because plants are sessile, this simple yet unique regulatory mechanism of TOR activation might be beneficial across their various ontogenic regimes.

## TOR signalling drives the cell cycle

In the budding yeast, two TOR genes are present which encode for the paralogs, Tor1 and Tor2, that make up the catalytic subunits of the TORC1 and TORC2 complexes, respectively, though TORC1 can also accommodate Tor2 at its active site (Loewith et al., 2002; Wedaman et al., 2003). Moreover, the structural aspects and composition of TORC1 and TORC2 are distinct, leading to rapamycin insensitivity in the latter (Loewith and Hall, 2011). On the other hand, in animals, only TOR, encoded by a single gene makes up the catalytic subunit of both mTORC1 and mTORC2 (Saxton and Sabatini, 2017). The two multimeric complexes vary in their overall subunit composition and therefore have specialized functions (Emmerstorfer-Augustin and Thorner, 2023). As in yeast, the mTORC1 in mammals and TORC1 in plants (hereafter TOR, since it lacks the TORC2 complex), control various aspects of cell cycle, growth and autophagy, while the TORC2/mTORC2 complexes regulate actin cytoskeleton dynamics (Schmidt et al., 1998; Cybulski and Hall, 2009; Emmerstorfer-Augustin and Thorner, 2023). Since the activity of TORC1/mTORC1/TOR complexes is controlled by nutrients, such as glucose and amino acids, and their regulation by these is well-known, therefore only their regulation in the cell cycle control will be discussed in the following sections.

**Figure 1:**
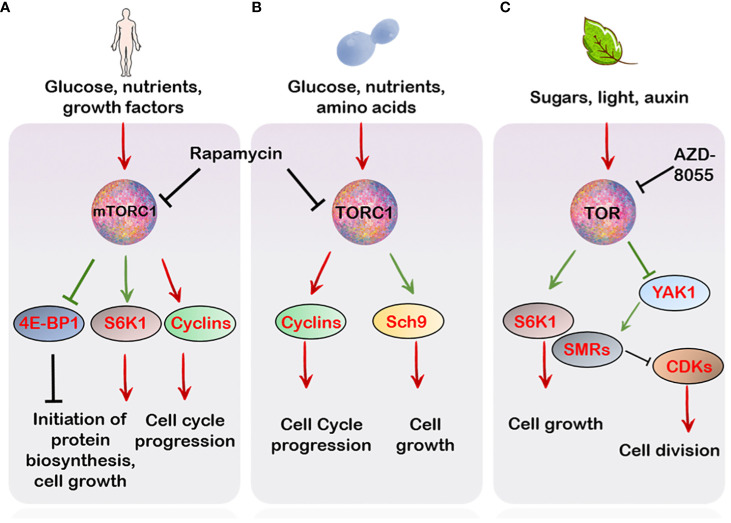
TOR regulation of cell cycle in different organisms. **(A)** In mammals, mTORC1-mediated signalling is relayed through the activation of its downstream effectors S6K1 and 4E-BP1 which regulate cell growth and cell cycle progression. Besides this, mTOR also controls the activation of various cyclins and CDKs which promote progression of different phases of the cell cycle **(B)** In *Saccharomyces cerevesiae*, the TORC1 favours progression of the cycle through activation of several G_1_ cyclins which activate various CDKs promoting the transition from G_1_ to S phase. Additionally Sch9, a major target of TORC1, which apart from controlling translation initiation and ribosome biogenesis also regulates entry into G_0_ (quiescence) **(C)** In plants, TOR, similar to the mammalian and yeast counterpart, controls phosphorylation events crucial for cell expansion and proliferation. This, in part, is mediated through S6K1 phosphorylation which integrates with the cell cycle machinery to favour growth over proliferation (see main text). Unlike S6K1, YAK1 is a negative regulator of growth. Phosphorylation by TOR inhibits YAK1’s activity which relieves the inhibition of CDKs by SMRs. Green arrows indicate direct phosphorylation by upstream targets.

Cell growth and cell proliferation are co-ordinately coupled processes which are regulated by the TOR kinase through its control over initiation of protein biosynthesis and ribosome biogenesis (Mahajan, 1994; Powers and Walter, 1999; Wullschleger et al., 2006). It is due to this property, in part, that necessitates TOR participation in the progression of the cell cycle. Additionally, the crucial role of TOR in this regard is exemplified by the action of the immunosuppressant drug rapamycin, (which specifically inhibits TORC1 and not TORC2) which results in a reduced rate of cell division, cell growth and cell cycle progression (Heitman et al., 1991; Barbet et al., 1996). In particular, TORC1 is crucial in maintaining cell cycle progression in the budding yeast, since its inhibition is lethal in the progression across all points in the cell cycle (Berset et al., 1998; Barbet et al., 2017). Specifically, the yeast TORC1 complex evokes both G_1_ to S and G_2_ to M phase transitions mostly by activation of various cyclins and de-repression of various CDKs (CYCLIN-DEPENDENT KINASES) (Heitman et al., 1991; Nakashima et al., 2008; Barbet et al., 2017) (**Figure 1**). Similarly, mTORC1 inhibition through rapamycin results in cell cycle arrest through repression of various cell cycle components including cyclins, CDKs as well reduced phosphorylation of the Rb (RETINOBLASTOMA) protein in various cell types (Hashemolhosseini et al., 1998; Decker et al., 2003; Gao et al., 2004; Balcazar et al., 2009; Jung et al., 2010) (**Figure 1**). Strikingly, many mammalian cancerous cells exhibit aberrant mTOR activation (Hsieh et al., 2012), suggesting a regulatory role of TOR in the tight regulation of cellular responses to environmental signals.

The support of TOR in the regulation of the plant cell proliferation and organ growth comes from the genetic screen of the TOR knockdown mutants and treatment of plant cells to TOR competitive inhibitors (Deprost et al., 2007; Montané and Menand, 2013). The TOR mutant displays various growth trait anomalies *viz*. reduced shoot and root growth, smaller rosette size, and reduced seed yield (Deprost et al., 2007), indicating TOR regulates plant fitness and life cycle traits. In line with this, pharmacological TOR inhibition through rapamycin treatment to the green alga, *Chlamydomonas* resulted in cell cycle arrest (Pérez-Pérez et al., 2017). Furthermore, supplementation of TOR inhibitors to Arabidopsis seedlings led to decreased overall meristem activity (Montané and Menand, 2013). In particular, the second-generation TOR inhibitor, AZD-8055 was shown to arrest Arabidopsis cell cycle specifically in the G_1_ phase in the roots (Desvoyes et al., 2020).

Beyond controlling cell division, TOR also controls cell size. Generally, cell growth is preceded by biosynthesis of macromolecules (Conlon and Raff, 1999; Polymenis and Schmidt, 1999). This is achieved through the activation of serine/threonine kinase, S6K1 (RIBOSOMAL PROTEIN S6 KINASE 1) signalling in eukaryotes (Thomas and Hall, 1997; Gingras et al., 2001; Magnuson et al., 2012). Moreover, phosphorylation of the S6K1 through TOR is frequently proposed as a readout of its activity (Brown et al., 1995; Schepetilnikov et al., 2013; Dobrenel et al., 2016) In mammals, the mTORC1 controls cell size through independent regulation of the downstream effectors, S6K1 and 4E-BP1 (eIF4E-BINDING PROTEIN 1) (Fingar and Blenis, 2004) (**Figure 1**). In *Drosophila*, yeast and mice, a reduction in both cell size and life span was observed upon S6K1 deficiency (Shima et al., 1998; Montagne et al., 1999; Sengupta et al., 2010). Particularly, the Sch9, S6K1 ortholog of yeast, regulates entry into G_0_ phase (quiescence) besides functioning to regulate ribosome biogenesis, protein synthesis and chronological life span (Fabrizio et al., 2001; Wang and Proud, 2006; Urban et al., 2007), hence pointing towards the conserved role of S6K1 in controlling cell size and ultimately proper growth and development (**Figure 1**). Of note, S6K1 deficiency mimics the effect of TOR inhibitors on cell size but not on cell proliferation, suggesting the regulation of cell size and proliferation can be uncoupled (Montagne et al., 1999; Ohanna et al., 2005; Dowling et al., 2010; Fumagalli and Pende, 2022).

**Figure 2:**
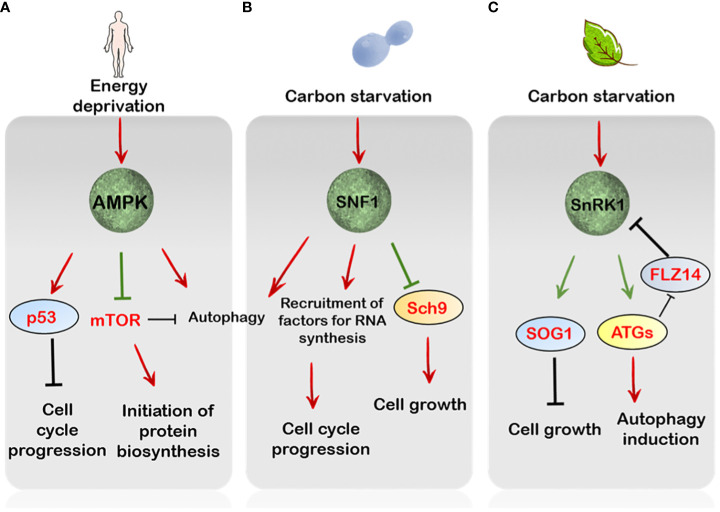
The regulation by AMP/SNF1/SnRK1 pathways of cell growth and proliferation. **(A)** In mammals, AMPK is essential to maintain genome integrity through phosphorylation of p53, leading to cell cycle arrest upon sensing DNA damage. Parallely, under starvation conditions AMPK overcomes mTORC1 signalling through RAPTOR phosphorylation. Besides this stress-triggered AMPK activation also induces autophagy **(B)** In Saccharomyces cerevisiae, under glucose limitation the SNF1 protein kinase modulates the expression of several factors crucial for DNA replication, repair and metabolism. Additionally, the Sch9 is inhibited by SNF1 under starvation conditions, thereby attenuating TORC1-mediated energy signalling. **(C)** In plants, SnRK1 mediates autophagy induction through activation of various ATG proteins. Furthermore, SnRK1 is activated through components of autophagy such as ATG8 by relieving its repression by the FLZ14 protein, which in turn controls the regulation of SnRK1 activity, hence forming a positive feedback loop. In addition, SnRK1 also controls cell growth and divisions through activation of the SOG1 protein under energy stressed conditions. Green arrows indicate direct phosphorylation by upstream targets.

In line with this, AtS6K1 also limits cell division and regulates growth in Arabidopsis (Henriques et al., 2010) (**Figure 1**). In the meticulous study by Henriques et al., 2010, AtS6K1 was shown to interact with both RBR1 and E2Fb. The S6K1 protein was demonstrated to be necessary for RBR1 localization into the nucleus where it suppresses E2Fb activity (Uemukai et al., 2005; Shimizu-Sato et al., 2008). Silencing of S6K1 resulted in cytoplasmic movement of RBR1 (RETINOBLASTOMA-RELATED 1) where it is phosphorylated via the CYCD3;1-CDKA1 protein complex (marker of the G_1_ and S phase) (Nakagami et al., 2002; Henriques et al., 2010). Interestingly, the suppression of AtS6K1 resulted in the reduction of cell size, increased ploidy levels and expression of CDKB1;1 (Henriques et al., 2010), a major marker of G_2_ to M phase transitions (**Figure 2**), suggesting that S6K1 negatively regulates cell proliferation (Henriques et al., 2010, Henriques et al., 2013).

A role of TOR signalling cascade is also evident in cellular differentiation in plant roots (Montané and Menand, 2013; Xiong et al., 2013). Recently, a downstream effector of TOR signalling, named AtYAK1 (for YET ANOTHER KINASE), an ortholog of the yeast YAK1, was shown to positively regulate the expression of the cell cycle inhibitors, SMRs (SIAMESE-RELATED) which repress CDKs activity, causing reduced meristematic activity and early differentiation along the root meristem, a phenotype also reminiscent of AZD-8055 treatment to roots (Montané and Menand, 2013; Barrada et al., 2019; Forzani et al., 2019) Moreover, AtYAK1 was also demonstrated to limit the expression of various cyclins along the root meristem, further implying AtYAK1 as a negative regulator of the cell cycle (Barrada et al., 2019). AtYAK1 is also directly phosphorylated by TOR, resulting in its inactivation (Forzani et al., 2019), suggesting that TOR through its direct control over YAK1, might control the progression from the G_1_ to S as well as G_2_ to M phase of the cell cycle (**Figure 1**).

In the light of above observations, TOR becomes all the more important in linking plant cell proliferation to organ growth, especially since TOR is highly expressed at the meristems (Menand et al., 2002). Plant meristems are marked by active cellular divisions and maintain stem cell population via self-renewal (Stahl and Simon, 2010). Because TOR is involved in cell proliferation, expansion and elongation through its induction of translation capacity, its participation is crucial in maintaining meristem activity in both shoot and root. Interestingly, differential activation of TOR kinase is observed across plant organs (Li et al., 2017). For instance, the synergistic effect of light and auxin activation of TOR is required to induce proliferation at the shoot apical meristem (SAM), while cell divisions at the root apical meristem (RAM) are merely glucose-TOR dependent (Xiong et al., 2013; Li et al., 2017), which makes good sense as shoots and roots are spatially separated and the former are the primary organs which are exposed to light. Hence, sugar-TOR signalling at the shoot apex is essential for transduction of these signals to the root, enforcing proper growth and development.

## AMPK/SNF1/SnRK1 mediate cell survival under stress

In eukaryotes various checkpoints govern the gatekeeping of progression of the sequential events of the cell cycle (Gorbsky, 1997; Velappan et al., 2017; Marescal and Cheeseman, 2020). Several checkpoints are also called upon during various stages of an organism’s normal growth and development as well as during times of stress (Qi and Zhang, 2019; Pedroza-Garcia et al., 2022). In this context, sugar availability plays an important role. For example, a drop in exogenous glucose levels resulted in the cell cycle halt as soon as in the G_1_ phase in animal cell cultures (Pardee, 1974). This glucose availability mediated arrest is also observed in case of yeast and plants (Jones et al., 2005; Hartig and Beck, 2006; Masuda et al., 2016), which demonstrates that sugars serve as conserved fundamental metabolites in regulating cell cycle checkpoints. In the presence of sufficient metabolic resources these checkpoints are bypassed to enable the organism to transfer error-free DNA to the next generation (Francis and Sorrell, 2001).

The halt in cell cycle progression is due, at least in part, to AMPK (in animals)/SNF1 (in yeast)/SnRK1 (in plants) pathways that harmonize growth in response to energy inadequacy, which is quite in line with their stress responsive roles (Jones et al., 2005; Gwinn et al., 2008). The AMPK/SNF1/SnRK1 are heterotrimeric proteins which serve as metabolic sensors to transduce signals activated upon energy starvation to regulate various transcriptomic and metabolic regimes and in this way promote cell survival (Hardie, 2007; Hedbacker and Carlson, 2008; Crozet et al., 2014; Emanuelle et al., 2015; Jamsheer K et al., 2021). Additionally, they control various aspects of normal growth and development including meiosis, aging and sporulation in yeast (Carlson et al., 1981; Ashrafi et al., 2000), normal cell cycle progression is mammals (Bettencourt-Dias et al., 2004; Lee et al., 2007; Dasgupta and Chhipa, 2016), and regulation of flowering time, circadian induction of gene expression in plants, to name a few (Wurzinger et al., 2018).

The SnRK family comprises of three subfamilies viz. SnRK1, and the plant specific SnRK2 and SnRK3 (Hey et al., 2010). The latter two subfamilies regulate several biological processes in response to various stresses (Halford and Hey, 2009). Since the AMPK/SNF1 proteins are more similar to the SnRK1 than to its other homologues, further discussion will be aimed at SnRK1 signalling-mediated regulation of the cell cycle. As in the other prototypes, the SnRK1 complex consists of one α catalytic and non-catalytic β and the hybrid βγ subunits (Gissot et al., 2004; Baena-González et al., 2007). Furthermore, each subunit type has many members, for instance, there are three variants of the α subunits (SnRK1.1/KIN10, SnRK1.1/KIN11 and SnRK1.3/KIN12) giving rise to many functional isozymes (Emanuelle et al., 2015).

AMPK has been linked to cell growth inhibition and the induction of autophagy (Hardie, 2011; Li and Chen, 2019). In mammals, glucose deprivation causes cell cycle arrest regulated by the AMPK-dependent mTOR inhibition and activation of the tumour suppressor, p53 (Inoki et al., 2003; Jones et al., 2005) (**Figure 2**). The AMPK has also been reported to directly control mTOR activity through phosphorylation of its components, implicating a regulatory control of mTOR activity by AMPK signalling, leading to cell cycle arrest (Gwinn et al., 2008) (**Figure 2**). Additionally, AMPK induces autophagy after detection of error in DNA synthesis (Mihaylova and Shaw, 2011; Szewczuk et al., 2020). The otherwise gratuitous induction of cell cycle through abnormal mTOR signalling is a key signature of cancerous cells (Inoki et al., 2003; Luo et al., 2010). Because DNA replication and subsequent cell proliferation are energy demanding processes, the activation of AMPK signalling to mediate a halt in the cell cycle progression is preeminent in this regard (Jeon and Hay, 2015).

The role of SNF1 signalling is also evident in controlling cell cycle progression since the yeast *snf1* mutants fail to arrest the cell cycle even under acute nutrient deprivation and additionally show reduced tolerance to heat shock (Thompson-Jaeger et al., 1991). In addition, SNF1 is also correlated with correct mitotic spindle assembly, suggesting it exerts positive role in cell division dynamics (Tripodi et al., 2018). This idea further supports the role of SNF1 in regulating cytokinesis through its interaction with other cell cycle components (Coccetti et al., 2018) (**Figure 2**). Besides this, SNF1 is also critical for the extension of chronological lifespan of yeast cells grown under caloric restricted environments, thus conferring longevity (Wierman et al., 2017; Maqani et al., 2018). More recently, a phosphoproteomic analysis has also demonstrated the ability of SNF1 to phosphorylate Sch9, the yeast S6K1, under nutrient limitation, impairing its phosphorylation by TORC1 and hence its activity (Caligaris et al., 2023). This indicates that SNF1 can antagonize the energy pathway through repression of the TOR-Sch9 axis under acute nutrient-deprived conditions (**Figure 2**).

In plants, the first functional evidence of SnRK1 signalling in the regulation of cell cycle was depicted through heterologous expression of rye, *Secale cereale* SnRK1 in the yeast system (Dickinson et al., 1999). As a result of SnRK1 overexpression, a reduction in cell size of yeast cells was noticed, probably because the cells exited too early from the cell cycle (Dickinson et al., 1999). Earlier, it was shown that the shuttling of AMPK/SNF1 to and from the nucleus is regulated through their association with distinct complexes, which can alter their biological functions depending upon substrate availability in different subcellular spaces (Hedbacker and Carlson, 2008; Afinanisa et al., 2021). In plants, such spatial regulation of TOR activity by SnRK1 was recently demonstrated to operate during ABA signalling in Arabidopsis root meristem (Belda-Palazón et al., 2022). In particular, under unstressed conditions, SnRK1 is sequestered in the nucleus through its interaction with ABA signalling components (Belda-Palazón et al., 2022). However, upon ABA perception, the translocation of the catalytic subunit of SnRK1 (KIN10) out of the nucleus results in the inhibition of TOR activity as KIN10 directly interacts with and phosphorylates RAPTOR, leading to attenuation of TOR activity and hence root growth (Nukarinen et al., 2016; Belda-Palazón et al., 2022). This indicates a considerable role of the interplay of SnRK1-TOR in the modulation of root system architecture through the control of cell growth dynamics.

A recent report has also suggested that SnRK1 might have a role in sensing DNA damage, eliciting what is known as the DDR (DNA Damage Response) (Hamasaki et al., 2019; Pedroza-Garcia et al., 2022) In essence, DDR comprises the structural changes in DNA which affects its replication and transcription and the ensuing mechanisms that come into play to preserve and protect genome integrity (Nakad and Schumacher, 2016). In plants, the transcription factor SOG1 (SUPPRESSOR OF GAMMA RESPONSE 1), similar to p53 in mammals with respect to function, lies at the heart of the DDR in response to various environmental stresses (De Schutter et al., 2007). Interestingly, SOG1 was identified to interact with SnRK1 catalytic subunits KIN10 and KIN11, in response to low energy (Hamasaki et al., 2019). Upon sensing low energy levels through ATP, SnRK1 was shown to phosphorylate SOG1 which then enhanced the expression of cell cycle-related genes in the hypocotyl including CYCA2s and CYCD3 to induce divisions rather than growth (Hamasaki et al., 2019) (**Figure 2**). Nevertheless, a direct and concrete link between SnRK1 activity and SOG1 mediated DNA repair is difficult to pinpoint and needs further exploration.

A vast body of evidence has also demonstrated the crosstalk between cell cycle regulation and autophagy, which is required for cell survival under metabolic pressures of general stress and energy starvation (Matsui et al., 2013; Chen et al., 2017; Marshall and Vierstra, 2018). Autophagy is the intracellular catabolic pathway of “self-engulfment” in which the cell breaks down its own organelles and cytosolic components upon perception of various stresses (Mathiassen et al., 2017). SnRK1, like its mammalian prototype, as a modulator of cell growth is also associated with autophagy in plants. This is exemplified by the induction of SnRK1 activity through KIN10 overexpression or downregulation of the TOR pathway components, both of which are shown to accelerate autophagy induction (Soto-Burgos and Bassham, 2017; Soto-Burgos et al., 2018). In yeast, TOR negatively regulates autophagy induction through regulatory control over the Atg1 (AUTOPHAGY-RELATED 1) protein complex, which consists of various other Atg proteins (Kamada et al., 2010). Under nutrient starvation, TORC1 activity is repressed resulting in the activation of the Atg13 (dephosphorylation), whose association with the Atg1 protein kinase is crucial for induction of autophagy (Kamada et al., 2010).Similarly, in plants, TOR knockdown lines exhibited constitutive autophagosome formation especially in the roots (Liu and Bassham, 2010), indicating the conserved role of TOR in autophagy repression. Conversely, TOR overexpression under nutrient limiting conditions restricted autophagy induction suggesting that stress-induced autophagy proceeds through repression of TOR activity (Pu et al., 2017).

Likewise, SnRK1 activation of autophagy is brought into action through both transcriptional and translational induction of *ATG* genes (Baena-González et al., 2007; Chen et al., 2017). Interestingly, the SnRK1-mediated induction of autophagy was reported to act upstream of TOR-mediated repression since a constitutive autophagy response was observed in the *kin10* loss-of-function mutants subjected to AZD-8055 treatment. Similarly, activation of TOR in KIN10 overexpressing seedlings subdued autophagy (Soto-Burgos and Bassham, 2017). A recent report by Yang et al., 2023 has depicted a crosstalk between SnRK1 and components of autophagy in Arabidopsis which might be conserved across the seed plants. In particular, SnRK1 induction of ATG8 activity through phosphorylation induces its interaction with the FLZ14 (FCS-LIKE ZINC FINGER) protein, thereby relieving SnRK1 repression by FLZ14 (Yang et al., 2023). Given that FLZ14 mediates SnRK1 inactivation under normal conditions, its overexpression in Arabidopsis responds poorly to carbon starvation (Yang et al., 2023). Essentially, this reveals a bi-directional flow of information between the SnRK1 signalling and the components of autophagy to induce appropriate responses under energy starved conditions (**Figure 2**).

## Sugar availability and TOR-SnRK1 crosstalk regulate cell cycle events in plants

### G_1_ to S phase

The typical cell division cycle in plants is categorized into four major phases: two gap/interphases (post-mitotic interphase G_1_ and the pre-mitotic interphase G_2_) that separate the M (mitosis) and S (synthesis) phases. CDKs are key regulators of cell cycle progression. CDKs bind to their regulatory subunits called cyclins to promote transitions through the cell cycle. The combinatorial and orderly participation of CDKs with cyclins is crucial for their activation and distinct phase progression (Vandepoele et al., 2002; Inagaki and Umeda, 2011; Sablowski and Carnier Dornelas, 2014). In plants various CDKs have been identified among which the CDKA;1 helps in both the G_1_ to S and G_2_ to M transitions, while the CDKBs mediate G_2_ to M phase transitions (Boudolf et al., 2004; Inzé, 2005; Nowack et al., 2012; Sablowski and Gutierrez, 2022). The CDKBs are suggested to be plant specific and their genesis can be traced back to some of the green alga, although red algae and other eukaryotic groups are also reported to possess B-type CDK activity (Corellou et al., 2005; Huysman et al., 2015). A similar diversity in the number of plant cyclins is also observed which bind the core CDKs and help mediate the transition through each phase of the cell cycle (Harashima and Schnittger, 2012). The A and B-type cyclins are closely related phylogenetically and are expressed during different intervals of the cell cycle (Wang et al., 2004; De Jager et al., 2005). More specifically, the A-type cyclins regulate nearly all the phases of the cell cycle while the B-type cyclins are preferential in their regulation of the G_2_ to M phase (De Jager et al., 2005).

The D-type cyclins are unique in their ability to be regulated via distinct environmental cues and regulate G_1_ to S phase progression (Meijer and Murray, 2000; Dewitte et al., 2007). Moreover, reports have also suggested the involvement of D-type cyclins during the G_2_ to M phase of the cell cycle (Kono et al., 2003). Previously, the effect of sugars in promoting G_1_ to S phase transition through the rate limiting factor, CYCD3;1, one of the major D-type cyclins in plants, had been suggested, indicating the link between sugar signalling and the initiation of cell cycle progression (Riou-Khamlichi et al., 2000; Menges et al., 2006). For instance, in starved Arabidopsis seedlings, the addition of metabolizable sugars was shown to induce the expression of *CYCD2* and *CYCD3* (Soni et al., 1995; Riou-Khamlichi et al., 2000). Interestingly, the expression of *CYCD3* was induced upon sugar supplementation and was not induced upon addition of cytokinin alone, suggesting sugar dependency on the activation of *CYCD3* lies upstream of hormonal control of *CYCD3* activation (Riou-Khamlichi et al., 2000). Similarly, sucrose responsiveness of *CYCD4;1* expression in regulating lateral root (LR) formation in Arabidopsis was shown to occur independently of the auxin-mediated LR induction (Nieuwland et al., 2009), further suggesting that multiple D-type cyclins are regulated independently by sugar signals.

**Figure 3:**
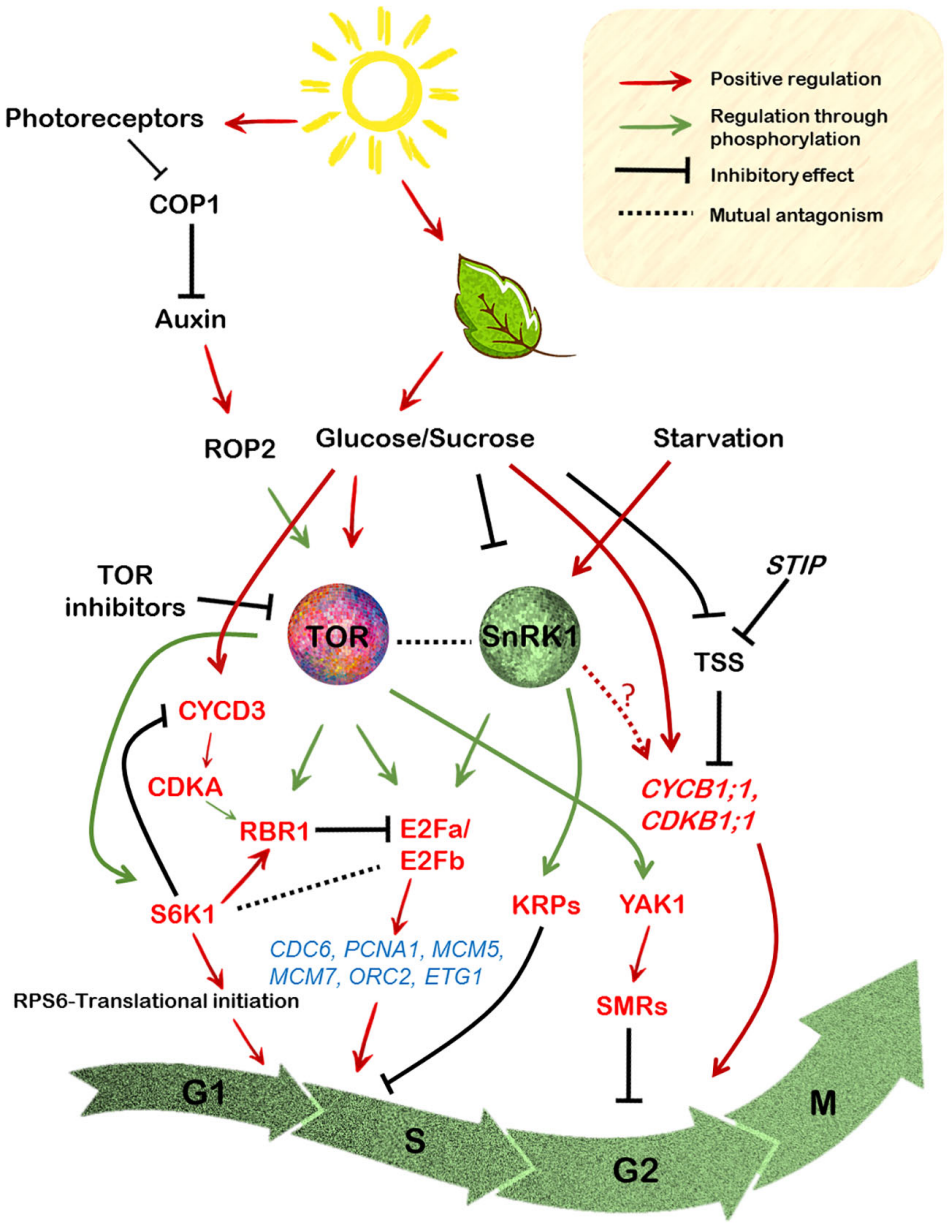
Regulation of the cell cycle components by integration of the sugar-TOR-SnRK1 signalling in the model plant Arabidopsis. Photosynthetically derived sugars drive various stages of the cell cycle. Glucose, the major end product of the light reactions activates TOR signalling which is repressed under starvation conditions by the SnRK1 signalling. Moreover, auxin activation of the ROP2 protein leads to its direct interaction with TOR, resulting in its phosphorylation, which further relays the signals to its downstream effectors like S6K1 and RPS6. The most common readout of TOR activity, the S6K1 protein however, exerts its effect on cell size rather than cell proliferation. S6K1 enhances cell growth and this is related to its RBR1 activating capacity through the promotion of its localization into the nucleus where it inhibits E2Fb protein activity. On the contrary the CYCD3;1-CDKA1 module is necessary for phosphorylation of RBR1, thereby promoting its retention in the cytoplasm. Sugar availability also leads to direct phosphorylation by TOR of the E2Fa/E2Fb proteins (N-terminal) that regulate the expression of various G1 to S phase marker genes encoding proteins required for DNA replication. E2Fa is repressed by hypophosphorylated RBR1 resulting in the inhibition of its transcription-inducing capacity. On the other hand, E2Fa is also directly phosphorylated (T314/T315) by the SnRK1 resulting in its degradation, the result of which is reduced transcription of these genes. Contrarily, SnRK1 also positively impacts cell cycle progression through direct phosphorylation of KRPs which abrogates their binding with cyclin/CDK complexes. Glucose-TOR signalling might also exert control over the G2 to M phase of the cell cycle through its interaction with the YAK1, which is involved in the suppression of various CDKs through activation of the SMRs. Moreover, metabolic sugars also directly control the expression of CYCB1;1/CDKB1 which is required for activation of cell division at the meristems.

The CYCD3;1 together with CDKA;1 mediate the inhibition of RBR1 protein through phosphorylation to facilitate the expression of genes required for the S phase entry (Weinberg, 1995; Meijer and Murray, 2000; Dewitte et al., 2007; Borghi et al., 2010) (**Figure 3**). The *Arabidopsis thaliana* RBR1, the only ortholog of animal Rb1, is crucial in controlling various events of cell division cycle ranging from progression into the cell cycle to stem cell maintenance, proliferation and differentiation (Borghi et al., 2010; Desvoyes and Gutierrez, 2020). Together with the E2F group of transcription factors, the E2F-RBR1 module plays a crucial role in determining cellular fate (Wildwater et al., 2005). In Arabidopsis, six E2F members are present (E2Fa, E2Fb, E2Fc, E2Fd/DEL2, E2Fe/DEL1 and E2Ff/DEL3) (Vandepoele et al., 2002). The binding of E2Fa, b and c to their recognition sequences are mediated by the dimerization partners DP (DPa and DPb) while DEL1/2/3 can act independently of DP proteins (Del Pozo et al., 2005). Furthermore, while E2Fa, b and c are considered as typical E2Fs, the latter three are atypical E2F proteins, since they lack transactivation and RBR1-binding domains (Sozzani et al., 2010). During G_1_ to S phase, RBR1 protein is hyper-phosphorylated via the CDKA;1-CYCD3;1 module (Hirano et al., 2008; Desvoyes et al., 2014), relieving its inhibitory effect on E2Fa/E2Fb and promoting S phase progression (Weinberg, 1995; Brehm et al., 1998; Desvoyes and Gutierrez, 2020) (**Figure 3**).

The ability of sucrose to mediate progression from the G_1_ to S phase is also RBR1 phosphorylation-dependent, failure of which results in cell cycle arrest in the Arabidopsis suspension cells (Hirano et al., 2011). This stems from the fact that sucrose starved cells exhibit decline in CYCD3;1 activity, leading to its subsequent degradation by the proteasome-dependent pathway, hence resulting in hypophosphorylation of RBR1 (Hirano et al., 2008, Hirano et al., 2011). RBR1 was also indicated to be a phosphorylation target of TOR probably through S6K1 as revealed in the quantitative phosphoproteomic analysis by Van Leene et al., 2019. In line with this, sucrose availability was also shown to enhance the phosphorylation of RBR1 at the conserved Ser807/811 sites, which is mediated by the CYCD3;1-CDKA1 protein module (Magyar et al., 2012). Furthermore, the interaction between the E2Fs and RBR1 was also shown to be mediated by sucrose (Magyar et al., 2012). Sucrose availability led to the formation of E2Fa-RBR1 dimer, while its depletion favoured E2Fb-RBR1 hetero-dimerization (Magyar et al., 2012).

E2Fs have been regarded as the key regulators of DNA synthesis in organisms (Dyson, 1998; Inzé and De Veylder, 2006). In essence, E2Fs bind to the promoter of various S phase specific genes viz. *ORC2,6* (ORIGIN RECOGNITION COMPLEX), *MCM3,5,7* (MINICHROMOSOME MAINTENANCE), *CDC6* (CELL DIVISION CYCLE 6), *ETG1* (E2F TARGET GENE) and *PCNA1* (PROLIFERATING CELL NUCLEAR ANTIGEN), which are involved in DNA replication (De Veylder et al., 2002) (**Figure 3**). Recent findings have suggested that E2Fa activity is largely regulated by glucose-TOR signalling. For instance, glucose-activated TOR kinase was shown to phosphorylate the N-terminal of the E2Fa protein in particular (Xiong et al., 2013; Li et al., 2017). Precisely, in shoots, both E2Fa and E2Fb proteins were shown to be the phospho-targets of TOR in the regulation of true leaf organogenesis, whereas in case of root meristem activation only E2Fa was demonstrated to be a TOR target, probably since E2Fa is predominantly expressed in the roots (Xiong et al., 2013; Li et al., 2017). Consistent with the role of E2Fa in maintaining cell cycle progression, the *e2fa e2fb* double mutants exhibited reduced expression of the S phase genes and a defective true leaf development pattern (Li et al., 2017). Furthermore, the single *e2fa* null mutant also displayed diminished expression of the S phase genes and a compromised root growth under glucose fed conditions (Xiong et al., 2013).

Interestingly, E2Fa has also been shown to be a target of SnRK1 signalling. In the most recent analysis of SnRK1 regulation of cell cycle, Son et al., 2023 demonstrated that SnRK1 impinges on E2Fa activity by directly phosphorylating it at T314/T315, causing its degradation and leading to suppression of cell proliferation and therefore restricted primary root growth. As discussed above, a concomitant decrease in the expression of various S phase marker genes was also observed (Son et al., 2023). Collectively, it can be noticed that E2Fa as a mutual target, acts like a switch for the TOR-SnRK1 duo in the control of G_1_ to S transition which is regulated through a differential impact on its activity (**Figure 3**).

Like in the mammalian counterpart, plants genes also encode for CDK inhibitors which upon physical contact cause the inhibition of their activity (Komaki and Sugimoto, 2012). Surprisingly, SnRK1 has been reported in the negative regulation of these inhibitors, implicating its role in the regulation of cell cycle progression in a positive light. In particular, both AtKRP6 and AtKRP7 (for KIP-RELATED PROTEINS) were shown to be directly phosphorylated by SnRK1 at Thr152 and Thr151, respectively (Guérinier et al., 2013). KRPs are associated with various CDKs through their binding ability, eventually affecting cell cycle progression (De Veylder et al., 2001; Van Leene et al., 2010). However, this post-transcriptional modification impaired their ability to bind and inhibit the CDK/cyclin complex, suggesting a promotive role of SnRK1 in the regulation of G_1_ to S phase transition (**Figure 3**).

### The G_2_ to M phase

Sugars have also been known to exert considerable influence over the G_2_ to M phase transition via regulating the activity and expression of mitotic cylins, especially CYCB1 (Riou-Khamlichi et al., 2000; Skylar et al., 2011). In plants, the progression into the M phase largely centres around the CYCB1;1 protein which was also the first B-type cyclin to be characterized in Arabidopsis (Colón-Carmona et al., 1999). Given that CYCB1;1 confers positive impact on cell division, various *cycb1* mutants display reduced root and shoot growth and seed yield (Motta et al., 2022), implicating CYCB1 as a major factor linking plant cell divisions to organ growth and development.

The main support for the involvement of sugar regulation of mitotic CYCB1;1 in plant growth is the observation that the glucose insensitivity of the *gig* (GLUCOSE INSENSITIVE GROWTH) mutants resulted in reduced cell divisions and therefore compromised growth of root meristem as monitored by *CYCB1;1::GUS* expression (Lee et al., 2012). Most strikingly, the finding that meristems of the *stip* mutants (for STIMPY) which are arrested in the G_2_ phase, can be substituted by metabolic sugars in their requirement for CYCB1;1 induction (Skylar et al., 2011), further confirms the involvement of sugar availability in the stimulation of both shoot and root meristem activity through cell proliferation. Interestingly, the meristem defects in the *stip* mutants were ascribed to the induction of high levels of the transcriptional repressor TSS (TPR-DOMAIN SUPPRESSOR OF STIMPY) which is responsible for the meristem cell division arrest (Skylar et al., 2011) (**Figure 3**). Metabolic sugars like glucose and sucrose and not sorbitol were able to rescue *stip* mutants of the TSS mediated repression (Skylar et al., 2011). Similarly, in maize, sugars, both glucose and sucrose were shown to regulate *ZmCYCB1;2* and *ZmCYCB2;1* expression upon seed germination (Lara-Núñez et al., 2021), indicating sugar induction of cyclins is prevalent in other plants too.

Glucose signalling was also demonstrated to confer transcriptional control over several cell cycle-related genes including those that are required for maintaining root meristem integrity (Xiong and Sheen, 2013; Xiong et al., 2013). In a very intriguing study by Li et al., 2017, glucose was reported to scavenge meristematic cells from mitotic quiescence. However, the mechanism by which glucose signalling activates this process is differentially regulated in shoot versus the root. In the shoot apex, both light and glucose act synergistically in inducing cell proliferation through stimulation of TOR activity, while glucose but not light was strictly necessary for inducing cell divisions in the root apex (Li et al., 2017). Furthermore, auxin was found to substitute for light but not glucose-dependency of TOR activation, suggesting that both photosynthetically derived sugar signals and auxin can induce cell cycle progression to activate divisions in the SAM (**Figure 3**). Furthermore, light-enhanced TOR induction of initiation of protein biosynthesis is also mediated by auxin signalling directly through the ROP2 GTPase (RHO OF PLANTS 2) (Schepetilnikov et al., 2017). Previously, ROPs have been reported to be auxin responsive, transducing the signals within very short time periods after the hormone treatment (Dubey et al., 2021). Because ROP2 positively affects TOR activity, the ablation of ROP2 activity results in reduced TOR signalling in the *rop2,4,6* mutant plants (Schepetilnikov et al., 2017). Furthermore, a recent study suggested that the TOR-ROP2 module is itself regulated by the light signalling protein COP1 (CONSTITUTIVE PHOTOMORPHOGENIC 1) (Cai et al., 2017), a negative regulator of light mediated responses (Ang et al., 1998) (**Figure 3**). Interesting, TOR through its downstream effector S6K1, also conveys signals from light through phosphorylation of the RPS6 (ribosomal protein S6), a key component of the 40S ribosomal subunit (Ruvinsky and Meyuhas, 2006) to augment initiation of translation in de-etiolating seedlings (Chen et al., 2018). Accordingly, a constitutive gain-of-function of RPS6 resulted in the induction of protein biosynthesis and an enhancement in cotyledon opening and development (Ren et al., 2012; Chen et al., 2018). Such interconnections are integral to promote leaf organogenesis at the shoot apical meristem (Ren et al., 2012; Pfeiffer et al., 2016).

Interestingly, in yeast, endogenous IAA production was demonstrated to downregulate TORC1 activity (Nicastro et al., 2021). The repression in TORC1 activity, as discussed above, leads to cell quiescence, hence this strategy of accumulation of high IAA during the stationary phase might prove beneficial in providing prolonged survival in this organism. Similarly, in an earlier report, auxin was shown to inhibit RPS6 (Snyder et al., 2019). This fact is in stark contrast to what is discussed above in case of plant system, in which auxin has been described as a well-known TOR activator. This diversity in auxin responses amongst different taxa of organisms stems from the capacity of the hormone to have dose-dependent effects (Leyser, 2018). Thus, it appears that the concentration-dependent role of auxin on TOR activity could regulate prioritization of cellular responses in different taxa.

Previously, a co-occurrence of CYCB1;1-CDKB1 expression and genotoxic stress has been reported, which points out the duality of these proteins in regulating not only mitosis but also DDR (Chen et al., 2003; Culligan et al., 2006; Biedermann et al., 2017). Interestingly, this upregulation in *CYCB1;1* transcript levels was shown to be mediated through the central DNA damage sensors kinase, ATM (ATAXIA TELANGIECTASIA MUTATED) and ATR (ATAXIA TELANGIECTASIA MUTATED and RAD3 RELATED) which are known to phosphorylate SOG1 (Culligan et al., 2006; Yoshiyama et al., 2013; Roitinger et al., 2015). Furthermore, reports have demonstrated that CYCB1;1 as a direct target of SOG1 might play a crucial role in DDR (Weimer et al., 2012, Weimer et al., 2016; Biedermann et al., 2017; Schnittger and De Veylder, 2018). In line with this, the CDKB1-CYCB1 complex was shown to be essential in mediating RBR1 nuclear localization during DNA damage where it recruits the RAD51 (RADIATION SENSITIVE 51), a core mediator of DNA repair to the damaged sites (Biedermann et al., 2017). Concomitant with the role of sugars in the regulation of root meristem activity through CYCB1;1 expression, it is likely that sugar-induced activation of TOR activity might act to regulate both cell division and repair mechanisms. However, the exact mechanism by which TOR might participate in this regard remains to be shown. Likewise, SnRK1 was also shown to be associated with transcriptional regulation of *CYCB1;1* (Krasnoperova et al., 2016). In particular, this study in Arabidopsis demonstrated the transcript levels of *CYCB1;1* to be diminished in loss-of-function mutants of KIN10/KIN11 resulting in a reduced mitotic index in the RAM under both normal and energy deficient conditions (Krasnoperova et al., 2016), though it remains unclear how this effect is linked to growth and development.

Clearly, the above examples suggest that TOR-SnRK1 signalling might not always act antagonistically in regulating the same process as depicted in the recent study by (Saile et al., 2023) wherein a concerted effect of TOR-SnRK1 mutualism was shown to induce hypocotyl elongation under the skotomorphogenic regime. Another study indicates the concurrent role of TOR and SnRK1 in fine-tuning stomatal development (Han et al., 2022). This harmony between the TOR-SnRK1 crosstalk has also been reviewed at length by Rodriguez et al., 2019. Such concerted roles are in contrast to their already well-known antagonism and reveal a mechanism by which antagonistic regulatory pathways can function simultaneously to promote key aspects of plant growth and development.

## Conclusion and future prospects

In summation, sugars appear to serve as a fundamental role in the cell cycle progression. The master regulators, TOR and SnRK1 co-ordinate several such processes in accordance with nutrient availability. Most often than not, the sugar mediated TOR-SnRK1 signal transduction pathways are considered to be governing mutually exclusive processes. Evidence has accumulated suggesting that these pathways might be coordinated to operate in the same pathway of growth and development such as discussed in case of the modulation of cell cycle events. This is also exemplified by various studies which do not fall under the scope of this review and are discussed in detail elsewhere. Combating future challenges would warrant identification of direct targets that connect TOR-SnRK1 signalling to gain a deeper understanding on how these central kinases affect several developmental processes. Particularly in plants, since much of the regulation of cell cycle progression and its modulation by essential factors are affected by TOR governed signalling, future prospects should be aimed at elucidating various facets of SnRK1-mediated pathway in the modulation of these events. Furthermore, quantitative phosphoproteomics might reveal potential targets of the TOR/SnRK1 enzymes as well as the putative phosphosites on these targets. Also, it will be interesting to decipher probable candidates that are intertwined in the regulation of cell cycle in plants beyond the model plant Arabidopsis. A complete overview of the cell cycle regulation in different organisms through combinatorial omics approach might be utilized in order to understand the shared ancestry of eukaryotic cell cycle and its regulatory factors in different taxa especially in the green lineage.

## Author contributions

SR: Conceptualization, Writing – original draft. AL: Writing – review & editing.
